# Socioeconomic inequalities in mental well-being among Hungarian adolescents: a cross-sectional study

**DOI:** 10.1186/s12939-014-0100-8

**Published:** 2014-10-28

**Authors:** Szabolcs Varga, Bettina F Piko, Kevin M Fitzpatrick

**Affiliations:** Institute of Behavioral Sciences, Semmelweis University, 1089 Nagyvarad sqr. 4, Budapest, Hungary; School of Ph. D. studies, Semmelweis University, 1085 Ulloi str. 26, Budapest, Hungary; Department of Behavioral Sciences, University of Szeged, 6722 Szentharomsag str. 5, Szeged, Hungary; Department of Sociology and Criminal Justice, University of Arkansas, 72701 Fayetteville, AR USA

**Keywords:** Socioeconomic status, Adolescents, Mental health, Mental well-being

## Abstract

**Introduction:**

According to several empirical studies, mental well-being is significant in adolescence; adolescent’s social network is undergoing radical changes while at the same time depression is increasing. The primary goal of our study is to determine whether socioeconomic status (SES) is associated with mental health status of Hungarian adolescents and the strength and nature of this association.

**Methods:**

Our sample was comprised of three high schools of Debrecen (the second largest city of Hungary). Data were collected in January 2013. In all, 471 students filled out the questionnaire from 22 classes (14–18 years old). ‘Absolute’ (education and occupational status of the parents, assessed by the adolescent) and ‘subjective’ (self-assessment of family’s social class) SES measures and five mental health indicators (shyness, loneliness, need to belong, psychosomatic symptoms, self-esteem) were involved. Descriptive statistics and binary logistic regression analyses were used to examine the relationships between family SES and mental health indicators.

**Results:**

Our results indicate that association between adolescents’ ‘subjective’ SES and mental well-being is not gradient-like. Manual employment and unemployment status of both parents also proved to be significant determinants of mental health status.

**Conclusions:**

According to our results, professionals of school-based mental health programs should consider students whose parents are unemployed or have manual occupational status as a high risk group in terms of mental well-being.

## Introduction

In the last 40 years, several studies have focused on the relationship between socioeconomic status (SES) and health across the life-course [1,2]. Research on this relationship among adults suggest that adults’ mortality and morbidity were significant and in a gradient relationship with a person’s level of education, occupational status, and income [3,4]. This relationship has been found to be significant in both European and American samples; however, the relationship between socioeconomic status (SES) and health is not consistent across the life course [5,6].

Previous empirical studies have demonstrated the importance of examining the relationship between social status and mental health [7–9]. Adolescent’s social network is undergoing radical changes while at the same time depression and the frequency of psychosomatic symptoms are increasing [10]. Despite being free of serious physical illness, many adolescents report subjective health complaints [11]. Frequent psychosomatic symptoms may also increase the risk of disturbed neuro-psychological development since somatization can be a learned reaction to stress and emotional traumas that may also be manifested in physical illness later on [12].

WHO proposed mental health as „…a state of well-being in which the individual realizes his or her own abilities, can cope with the normal stresses of life, can work productively and fruitfully, and is able to make a contribution to his or her community” [p.12] [13]. Well-being is more than the absence of mental illness and can be measured by several psychological and social indicators (e.g. life satisfaction, depression, anxiety, self-esteem, etc.) [14,15]. Adolescents’ low mental health may lead to illnesses such as depression and social phobias [16] and may have major implications for adult morbidity and mortality [17].

From the 1960s, until 1989–90, the shifting of the economic system, and the living standard in Hungary was relatively high compared to other East-Central European countries, with lower rates of social inequalities. After this change, socioeconomic inequality increased rapidly and a new meaning of class appeared [18]. After 1990, the development of a market economy led to important differences between the social classes in several dimensions (income, lifestyle, health, education etc.). Unemployment was also a new phenomenon for Hungarians to face. Accordingly Hungary, as an East-Central European post-socialist country, is a special and unique social environment to study from a socioeconomic perspective [10].

Several studies have found substantive correlations between low family SES and low mental health among adolescents [10,19,20]. These studies use self-administered questionnaires to measure adolescents’ family SES. Psychosomatic symptoms like headache, backache, nervousness, sleeping difficulties and tiredness have been found to be significantly related to both ‘subjective’ (self-assessment of family’s social class) and ‘absolute’ (occupation and education of parents and family structure, assessed by the adolescent) measures of SES [6,17]. Adolescents’ shyness is also related significantly to these family SES indicators: a relatively higher percentage of shyness occurs among students from lower socio-economic classes [21]. Students’ need to belong moderately correlates with psychosomatic symptoms and shyness [22]. Self-esteem is positively related to family affluence of the adolescents [23]. Thus, family socioeconomic status is strongly linked with several dimensions of mental health and differences across a wide range of demographic groups, varying by age, gender and different SES measures [24].

Given these results, we believe it is important to measure adolescents’ mental well-being using a multidimensional approach that includes five indicators. *Psychosomatic symptoms*, *self*-*esteem* and *loneliness* have been used as indicators of adolescents’ mental well-being [10,23,25]. *Shyness* is also related to a variety of adverse psychological health outcomes (depression, social phobias, psychosomatic symptoms, loneliness, etc.) [26], and have been used as a mental health indicator among adolescents in past studies [27,28]. In addition, belongingness has been found to be an essential predictor of mental well-being [29]. Social rejection of a student is often linked with development of greater *need to belong* [30]. If need to belong is not satisfied, feeling of loneliness increases, which in turn, has a negative effect on the mental well-being of adolescents [31]. We have decided to use indicators that include both social and individual aspects of mental well-being. This type of measurement is particularly relevant to consider since most adolescents during this age period are free of serious physical illness, yet they report considerable psychosomatic and psychological distress symptomatology [10].

The goal of our study is to determine whether family SES is associated with mental health status in a sample of Hungarian adolescents. Studies have measured family SES in a number of different ways. ‘Absolute’ variables have been used, including parents’ education [32–34], occupational or employment status [6,10,32], family income and affluence [35,36], assessed by the adolescent. In addition, ‘subjective’ SES variable was included in several studies, based on the self-assessment of the adolescents about the class of his/her own family [6,10]. We attempt to describe the strength and pattern of this association by multidimensional measures of family SES with self-administered questionnaires, including both ‘absolute’ (educational level and occupational status of both parents, assessed by the adolescents) [14,37–39] and ‘subjective’ (asking the students to rate their own families’ socioeconomic status) [10,14,40] variables. We use the SES related terminology of McLaughlin et al. [41]. According to previous studies, we expect a positive correlation between both SES measures and mental well-being, and expect that ‘subjective’ SES will act as a more protective mechanism compared to ‘absolute’ indicators [6,10,33,41].

## Methods

### Participants and procedure

Our sample contains data from 14–22 year-old adolescents representing three different types of secondary school trainings, including: a technical college, vocational education, and a grammar school track from three high schools of Debrecen, the second largest city in Hungary. The three high schools in the sample are from three different districts and represent different qualities of education. The study involved an elite grammar school from the city centre, a technical college, and a relatively low-quality vocational school from the suburban region. The first version of the questionnaire was tested in one high school class to explore the ’weak points’, which are not unequivocal for the students. After the test, the questionnaire was reviewed and revised based on the feedback from students. The questionnaires were completed during January, 2013. Of the 503 possible eligible students from 22 classes, 501 students completed the survey (99.6% response rate); only two adolescents refused to participate in the study. In each class graduated teachers distributed the questionnaires to adolescents with as set a brief instructions. Teachers were instructed about the essential information they should tell the students: participation was confidential and voluntary, the goal of the study, and some technical information about the questionnaire. Students completed the questionnaires during a single class period. The schools and classes in the sample were selected randomly. The Institutional Ethical Boards and principals of all participated high schools approved the questionnaire and method of the study in December 2012 and January 2013. For the purpose of this study we excluded those aged above 18 years giving a total sample size of 471 high school students (ages 14–18; mean = 16.21 years, SD = 1.13 years; 33% boys). Boys were underrepresented in the sample due to our sampling method (We acknowledge that randomization may lead to some imbalance of the sample).

### Measurement

In the current study, *socioeconomic status* was measured in the questionnaire, including both ‘absolute’ and ‛subjective’ variables. ‘Absolute’ measures were based on the educational level and occupational status of both parents, assessed by the students [14,37–39]. Originally, educational level was classified to include four levels: primary education, apprenticeship, General Certificate of Education, and university or college degree. Occupational status included six categories: skilled non-manual and managerial, other non-manual, self-employed, skilled manual, unskilled manual, and unemployed. Given our intent in the current analysis and for comparison with previous studies, educational level was recoded into ‛high school or below’ (primary education, apprenticeship and General Certificate of Education) and ‛college/university’; occupational status was recoded into ‛non-manual’ (skilled non-manual and managerial, other non-manual), ‛self-employed’ , ‛manual’ (skilled and unskilled manual) and ‛unemployed’ categories. We also included a ‘subjective’ SES indicator, asking the students to rate their own families’ socioeconomic status [10,14,40]. Five possible answers were included (lower, lower-middle, middle, upper-middle and upper class); these five categorical responses were recoded into three categories (low/lower-middle, middle and upper/upper-middle class).

*Mental health* was measured using five different scales that tapped into shyness, the desire/need to belong, psychosomatic symptoms, loneliness, and self-esteem. All of these scales have been widely used and adopted in Hungarian populations [17,42]. The questionnaires were translated and back-translated by two independent experts.

We measured shyness using the Revised Cheek and Buss Shyness Scale (RCBS) [26,43]. The scale contained 13 items (e.g., “it is hard for me to act natural when I am meeting new people”), including 4 reversed items (e.g., “It does not take me long to overcome my shyness in new situations”). Answers were coded from 0 to 4 (4 to 0 for reversed items), with the response categories ranging from “Not at all” to “Entirely agree” based on the level of agreement. Based on the current data it was reliable with a Cronbach’s alpha of 0.78, with a mean score of 16.4 (SD = 8.4).

The Need to Belong Scale was developed to measure “the desire for acceptance and belonging for use in an experiment investigating reactions to potential acceptance and rejection.” [p. 2.] [22]. The scale contained 10 items (e.g., “I try hard not to do things that will make other people avoid or reject me”), including 4 reversed statements (e.g., “If other people don't seem to accept me, I don't let it bother me”) with the same response categories. Answers were coded from 1 to 5 (5 to 1 for reversed items). Based on our data the scale’s Cronbach’s alpha was 0.60, with a mean score of 33.9 (SD = 5.7).

We measured loneliness using the revised form of UCLA Loneliness scale [44], which contains 20 items (e.g., “I have nobody to talk to”), including 10 reversed items (e.g., “I can find companionship when I want it”). Rosenberg’s Self-esteem scale [45], containing 10 items (e.g., “I feel that I am a person of worth, at least on an equal plane with others”), including 5 reversed items (e.g., “I certainly feel useless at times”) was applied to measure students’ self-esteem. The loneliness scale was coded from 1 to 4, the self-esteem scale from 0 to 3 (opposite in both scales’ reversed items). The scales yielded the mean scores of 32.1 (SD = 8.1) for the UCLA Loneliness scale and 19.1 (SD = 6) for Rosenberg’s Self-esteem scale. Both scales have 4 response categories from “Not at all” to “Entirely agree” and were reliable with Cronbach’s alpha coefficients of 0.85 (loneliness) and 0.83 (self-esteem).

Finally, the Psychosomatic Symptom Checklist [46] was used to assess the frequency of adolescents’ reporting of psychosomatic symptoms. Each item on the list focuses on a single symptom, such as headache, sleeping disorder, stomachache, etc. The items were coded from 1 to 4, with the response categories of “never”, “rarely”, “sometimes” and “often”. Cronbach’s alpha was 0.77 for the current sample. All the scales mentioned above were negatively related to adolescents’ mental well-being (higher score means lower mental well-being), excluding Rosenberg’s Self-esteem scale, which is positively related to mental health. Based on the scales, two groups were identified: those who have high and those who have low levels of shyness, loneliness, etc. (above and below the median value).

### Statistical methods

Statistical analysis was conducted with SPSS for MS Windows Release 15.0 software, with a significance level of 0.05. The first part of the data analysis examines the descriptive statistics for all included dependent and independent variables. The main focus of the analysis was to examine the relationship between family SES measures (as independent) and mental well-being (as dependent) variables using binary logistic regression. The results of the binary logistic regression analyses are presented as a series of odds. The baseline odds are set to 1.0. An odds ratio >1.0 indicates that there is a positive association between the factors of interest to the baseline odds while a value <1.0 indicates the inverse. Confidence intervals (95%) were also calculated for significant relationships (depending on the criterion that the intervals do not contain a value = 1.0). Strength of an association was also assessed by the odds ratio. Mutually adjusted models are presented.

## Results

Socio-demographic, socioeconomic and mental well-being characteristics for the high-school student sample are presented in Table 1. The majority of adolescents considered themselves middle class (61.1%), a total of 17.6 percent said they belong to upper or upper-middle class and only 12.6 percent thought they were in a lower or lower-middle class family. Seventy percent of the students’ fathers and 65.7 percent of their mothers had high school or less than a high school education. With regards to occupation, 20.8 percent of fathers worked in non-manual jobs, 37.1 percent manual jobs, 24.6 percent of them were self-employed and 6 percent were unemployed. On the other hand, adolescents’ mothers’ non-manual status was the most frequent category (36.1%); more than a quarter of students (26.5%) reported that their mothers had a manual occupation, 15.2 percent of mothers were unemployed and 13.2 percent were self-employed. Similarly to the rates of Hungarian secondary education [47], majority of the surveyed adolescents study in grammar school or vocational education and only the minority are at a technical college. Even so technical college students were underrepresented in the sample.

**Table 1:** **General characteristics of Hungarian high school students (n = 471)**

**Characteristics**	**%**	**Mean**	**SD**	**N (response rate)**
*Absolute SES*				
**Father:**				
Schooling				450 (95.5%)
High school or below	71.8%			338
College/university	23.8%			112
Occupation				418 (88.7%)
Non-manual	20.4%			96
Self-employed	24.2%			114
Manual	38.2%			180
Unemployed	5.9%			28
**Mother:**				
Schooling				454 (96.4%)
High school or below	66.2%			312
College/university	30.1%			142
Occupation				430 (91.3%)
Non-manual	35%			165
Self-employed	13.4%			63
Manual	26.8%			126
Unemployed	15.9%			75
*Subjective SES*				428 (90.9%)
Lower class	1.5%			7
Lower-middle class	11%			52
Middle class	61.1%			288
Upper-middle class	15.5%			73
Upper class	1.7%			8
*Socio*-*demographics*				
Gender				470 (99.8%)
Male	32.9%			155
Female	66.9%			315
Age		16.2	1.1	471 (100%)
Type of school				471 (100%)
Grammar	51.2%			241
Modern	42.9%			202
Technical	5.9%			28
*Mental health*				
Need to belong		34	5.7	440 (93.4%)
Loneliness		32	8	402 (85.4%)
Psychosomatic symptoms		14.8	4.3	450 (95.5%)
Self-esteem		19	6.1	437 (92.8%)
Shyness		29.5	8.3	395 (83.9%)

Percentages do not add up to 100% in most cases due to some missing values and rounding.

Table 2 presents the results of the binary logistic regression analyses, including odds ratios (OR) for the relationship between family SES indicators, gender and age (independent), and the five mental health (dependent) indicators. This type of regression analysis focuses on the importance of each independent variable and their contribution to differences in the odds in adolescents’ mental well-being.

**Table 2:** **Logistic regression estimates (OR) of predictors of mental well-being indicators [OR (95% CI)] (n = 471)**

**Predictor variables**	**High level of need to belong**	**High level of loneliness**	**High level of psychosomatic symptoms**	**High level of self-esteem**	**High level of shyness**
Father’s schooling					
College/University degree^a^	1	1	1	1	1
High school level or below	0.9 (0.6 – 1.4)	1.7 (1.1 – 2.7)*	1.4 (0.9 – 2.0)	0.6 (0.4 – 0.9)*	1.5 (1.0 – 2.3)
Mother’s schooling					
College/University degree^a^	1	1	1	1	1
High school level or below	0.9 (0.6 – 1.4)	1.1 (0.7 – 1.6)	0.9 (0.6 – 1.3)	0.7 (0.4 – 0.9)*	1.7 (1.1 – 2.6)**
Father’s employment status					
Non-manual^a^	1	1	1	1	1
Self-employed	1.3 (0.7 – 2.2)	1.1 (0.6 – 2.0)	1.6 (0.9 – 2.8)	0.8 (0.5 – 1.4)	0.9 (0.5 – 1.6)
Manual	1.1 (0.7 – 1.9)	1.6 (1.0 – 2.8)	1.9 (1.1 – 3.2)*	0.4 (0.3 – 0.7)***	2.3 (1.3 – 3.9)**
Unemployed	3.0 (1.2 – 7.7)*	2.8 (1.1 – 7.6)*	1.2 (0.5 – 2.8)	0.4 (0.2 – 0.9)*	1.6 (0.6 – 4.1)
Mother’s employment status					
Non-manual^a^	1	1	1	1	1
Self-employed	0.7 (0.4 – 1.2)	0.7 (0.4 – 1.4)	0.8 (0.4 – 1.4)	0.8 (0.5 – 1.5)	0.8 (0.4 – 1.5)
Manual	0.8 (0.5 – 1.2)	1.1 (0.7 – 1.8)	1.0 (0.6 – 1.5)	0.4 (0.2 – 0.6)***	1.8 (1.1 – 2.9)*
Unemployed	0.9 (0.5 – 1.6)	2.2 (1.2 – 4.0)*	1.4 (0.8 – 2.4)	0.5 (0.3 – 0.8)**	3.4 (1.8 – 6.5)***
Subjective SES					
Upper/upper-middle class^a^	1	1	1	1	1
Middle class	0.5 (0.3 – 1.0)	4.2 (2.0 – 8.8)***	2.0 (1.0 – 3.8)*	0.1 (0.1 – 0.3)***	2.8 (1.4 – 5.7)**
Lower/lower-middle class	0.6 (0.4 – 1.1)	2.3 (1.4 – 3.9)**	1.4 (0.9 – 2.4)	0.3 (0.2 – 0.6)***	2.3 (1.4 – 4.0)**
Gender					
Male^a^	1	1	1	1	1
Female	2.1 (1.4 – 3.1)***	0.9 (0.6 – 1.3)	2.6 (1.8 – 4.0)***	0.3 (0.2 – 0.5)***	1.1 (0.8 – 1.7)
Age (cont.)	0.9 (0.8 – 1.0)*	1.2 (1.0 – 1.4)*	1.0 (0.9 – 1.2)	1.2 (1.1 – 1.4)*	1.0 (0.8 – 1.1)
Type of school					
Technical college^a^	1	1	1	1	1
Vocational education	0.5 (0.2-1.1)	0.9 (0.4-2.2)	0.7 (0.3-1.6)	3.2 (1.2-8.2)*	1.2 (0.5-3.3)
Grammar school	0.8 (0.4-1.7)	0.5 (0.2-1.6)	0.6 (0.3-1.3)	3.9 (1.5-9.9)**	0.5 (0.2-1.3)

^a^Reference category; *p <0.05; **p < 0.01; ***p < 0.001.

‛Subjective’ SES was the most influential indicator on adolescents’ mental health, four out of five well-being variables showed statistically significant relationships with this indicator. On the other hand, the relationships were not gradient-like. Both middle-class and lower/lower-middle class groups showed higher ORs of high level of loneliness (OR = 4.2; 95% CI = 2.0-8.8 and OR = 2.3; 95% CI = 1.4-3.9) and shyness (OR = 2.8; 95% CI = 1.4-5.7 and OR = 2.3; 95% CI = 1.4-4.0), and lower odds of high self-esteem (OR = 0.1; 95% CI = 0.1-0.3 and OR = 0.3; 95% CI = 0.2-0.6) compared to upper/upper-middle class, but middle class group had the worse ORs (the most increased risk of high loneliness and shyness, and the lowest of high self-esteem).

Father’s occupational status played the most significant role in adolescents’ mental well-being among the ‛absolute’ SES indicators. Unemployment and manual status were influencing mental health in a negative way; father’s unemployment was related to higher loneliness (OR = 2.8; 95% CI = 1.1-7.6) and need to belong (OR = 3.1; 95% CI = 1.2-7.7), and manual employment status was related to higher levels of shyness (OR = 2.3; 95% CI = 1.3-3.9), psychosomatic symptoms (OR = 1.9; 95% CI = 1.1-3.2), and low self-esteem (OR = 0.4; 95% CI = 0.3-0.7) compared to the non-manual group. Relationship between well-being and mother’s employment status reflects the same pattern, but it was statistically significant in fewer categories. Mother’s unemployment may contribute to higher levels of loneliness (OR = 2.2; 95% CI = 1.2-4.0) and shyness (OR = 3.4; 95% CI = 1.8-6.5), and low self-esteem (OR = 0.5; 95% CI = 0.3-0.8). Manual occupational status of adolescent’s mother may decrease the risk of high self-esteem (OR = 0.4; 95% CI = 0.2-0.6) and increase the likelihood of higher levels of shyness (OR = 1.8; 95% CI = 1.1-2.9). Parents’ schooling was also related to mental well-being of adolescents. Lower levels of schooling of both parents were related to lower levels of self-esteem [OR = 0.6; 95% CI = 0.4-0.9 (father’s schooling) and OR = 0.7; 95% CI = 0.4-0.9 (mother’s schooling)] for students. In addition, the lower schooling of the mother increased the likelihood of shyness (OR = 1.7; 95% CI = 1.1-2.6) for students; father’s schooling was associated with higher levels of loneliness (OR = 1.7; 95% CI = 1.1-2.7).

Both age and gender were related to student mental well-being. Adolescents’ age showed positive relationships with loneliness and self-esteem, and an inverse relationship with the need to belong. Female students had higher risks for three mental well-being dimensions (higher ORs for level of need to belong and psychosomatic symptoms, lower OR for high self-esteem). Type of school was significantly related only to Rosenberg’s self-esteem scale. Adolescents have higher level of self-esteem from both vocational education (OR = 3.2; 95% CI = 1.2-8.2) and grammar school (OR = 3.9; 95% CI = 1.5-9.9) compared to technical college students.

## Discussion

The ‛subjective’ SES in our data shows similar rates compared to a national survey conducted in 2010 among Hungarian adolescents [42]. Among 9th and 11th grade students the majority were middle-class members (59.3% and 65.6%), and the second largest group was upper/upper-middle class (34.3% and 24.7%), similarly to our results. Eleven to twenty-seven percent of the fathers have a college/university degree. This rate is higher in the case of mothers (14.7%-30.8%) in every studied region. These rates are also similar to our results. Rosenberg’s self-esteem scale was used with the same average (18.86) and high reliability (0.83) in the national sample. Another national study [10] used the Psychosomatic Symptom Scale, with similar mean score (12.8) and high reliability coefficient (0.75).

Our results suggest that ‛absolute’ SES indicators may play a very limited role in adolescents’ mental health; only manual and unemployment status is associated with some mental well-being indicators. Meanwhile the ‛subjective’ SES indicator significantly correlates with four of five mental well-being scales, but this association is also not gradient-like. These findings support previous results, which suggest that ‛subjective’ SES is a relatively stronger predictor of mental health than ‛absolute’ measures [10,48]. The association between ‛subjective’ SES and mental well-being does not appear to be linear, because middle class groups actually had the highest risk of low mental well-being. Interestingly, previous findings suggest that adolescents that considered themselves to be mostly middle or lower class (as compared to those from upper/upper-middle classes) reported a higher likelihood of psychosomatic and depressive symptoms in a gradient-like way [6]. A possible explanation could be, those middle class adolescents’ higher aspirations and expectations for future social mobility as compared to upper class students (who have higher self-esteem for the future aspirations) or lower class students (who accept their status instead of high aspirations) [49–51]. All in all, more research is needed to detect the motives behind this finding.

Among ‛absolute’ indicators, father’s employment status was the less inconsistent predictor of student mental well-being. Students with manual worker or unemployed fathers have significantly higher odds of self-reporting low mental well-being, but the results were still limited and inconsistent. Mother’s occupation shows the same pattern, with even less consistency. Parents’ education was the worse predictor of adolescents’ mental health; these results partially support previous findings [6,10]. For example, a statistically significant positive association was found between adolescents’ mental health and the education of the parents. Among occupational status indicators, the mother’s status played a more influential role in determining the students’ mental health [6], but in our study, the father’s occupational status seemed to be slightly more important. According to our results both parents’ manual occupational status may increase the risk of some aspects of poorer mental well-being, similar to a previous research [6]. On the other hand, parents’ unemployed status was not clearly established as a negative predictor of adolescent psychosocial health. Although the negative role of father’s unemployment is well established, earlier studies from this region suggest that mother’s unemployment has a positive influence on adolescents’ psychosocial health, because of the substantial overlaps between the status of an unemployed women and a housewife. An unemployed women becoming a housewife can have a positive role in her children’s psychosomatic health [10]. Other studies established that mothers’ inactive status may contribute to their children’s increasing psychosomatic and depressive symptoms, mentioning attitudinal and the lack of financial resources as possible explanations [6]. Our results support this association in the case of mental well-being. During the period of socialism, the majority of Hungarian women were full-time employees, but with the dramatic economic shifts, an increasing number of them became full-time housewives [10]. We believe that during the recent economic crisis this situation has changed and there now appears to be a greater need for both parents’ to be working than ever before. However, it is certainly more difficult for these full-time housewives to return to the labor market—this might be a possible explanation for our results. In relation to this, a recent paper also suggests that children with unemployed parents usually have more health problems [52]. All in all, the problem of “being housewives or not” is more complex and there may be several other factors, e.g., control and support influencing it, therefore, further research is needed for clarification.

In the case of age and gender, previous studies found statistically significant relationships with adolescents’ psychosocial health, psychosomatic symptoms, and depressive symptoms [10,17]. Our study (with several limitations and inconsistency) supports these earlier reported relationships.

## Conclusions

Overall, our results suggest the following: (1) Occasionally positive, but non gradient-like and inconsistent association between adolescents’ family SES and mental well-being; (2) ‛Subjective’ SES was a better, but also inconsistent predictor of adolescents’ mental health compared to ‘absolute’ SES measures; (3) Among occupational statuses, only manual employment and unemployment of both parents correlated with some aspects of mental well-being; (4) Parents’ education was the weakest predictor among family SES variables; and (5) Both gender and age were significantly correlated with mental well-being in adolescence.

This population-based study may lead to a deeper and more differential understanding of the relationship between mental well-being and socioeconomic factors of adolescents in post-socialist countries. Our research focuses on a less frequently studied age group. Among health related researchers, only a few have focused on adolescents [1,2]. Another strength of our study is the multidimensional measurement of mental health, including five separate indicators. Statistically significant associations were found between four of five indicators and minimally two of the family SES measures. These indicators all had moderately high internal consistency (>0.77). Furthermore, family SES was measured multidimensionally in attempt to capture families’ social status differentially and extensively.

While there are a number of strengths to this study, we should note several limitations. We cannot provide a cause-and-effect relationship, because our study is cross-sectional. In addition, we used self-reported data on adolescents’ socioeconomic status, without any objective source and mental health, or without any clinical diagnoses. Among mental well-being measures the need to belong scale has a reliability coefficient of 0.6 in our data, and statistically significant association with only one family SES indicator (fathers’ unemployment). Accordingly we suggest that this scale, although widely used with high reliability internationally [22], needs further adaptation and validation to measure mental well-being among adolescents in Hungary. Finally, our findings may have limited generalizability, because of the study’s specific cultural context, sample size, and imbalance of the sample (only Hungarian adolescents were surveyed in one town, boys and technical college students were underrepresented comparing to the Hungarian adolescent population).

More research needs to focus on the context of mental health and family SES in population based studies, using different non-western societies to map features of these associations [53–58]. More extensive studies, using a longitudinal design should be conducted for a better understanding of progresses and cause-and-effect associations. In addition, other indicators should be considered in determining what precisely is accounting for variation in mental health status among adolescents, e.g., social network, lifestyle, social support and familial factors.

These findings support previous results [14], which emphasize the need for intervention to reduce poverty and social inequality among adolescents. Growing social inequalities can strengthen mental health disadvantage of low SES youth in post-socialist countries. In addition, we emphasize the importance of mental health promotion programs for adolescents, particularly for socially disadvantaged groups of adolescents. Based on a World Health Organization publication, effectiveness of a wide range of exemplary mental health promotion programs and policies seem justified. Their outcomes show that mental health promotion is a realistic option within a public health approach across e.g., parental care, schools and work settings. Effective and well-designed interventions can contribute to better mental health and well-being of the population, especially of adolescents in many fields of life [59]. In addition, the association between mental and physical health is widely recognized, they share many of the same social, environmental and economic determinants [60]. Accordingly, mental health promotion is an inseparable factor of health promotion in adolescence [61]. Promoting mental health in this age group also has a positive effect on future physical health status, like in the case of children [62]. According to our results, health policy may want to think about paying more attention to these adolescents who have parents with manual occupational status or who are unemployed. We also recommend professionals of school-based mental health programs to consider these adolescents as a high risk group with potentially low mental well-being and pay special attention to high schools of socio-economically disadvantaged regions.
